# Garcinol A Novel Inhibitor of Platelet Activation and Apoptosis

**DOI:** 10.3390/toxins11070382

**Published:** 2019-07-01

**Authors:** Hang Cao, Abdulla Al Mamun Bhuyan, Anja T. Umbach, Ke Ma, Oliver Borst, Meinrad Gawaz, Shaqiu Zhang, Bernd Nürnberg, Florian Lang

**Affiliations:** 1 Department of Pharmacology & Experimental Therapy, University of Tübingen, 72074 Tübingen, Germany; 2 Department of Cardiology and Cardiovascular Medicine, University Hospital of Tübingen, 72076 Tübingen, Germany; 3 Institute of Preventive Veterinary Medicine, Sichuan Agricultural University, Wenjiang, Chengdu 611130, China; 4 Department of Vegetative & Clinical Physiology, University of Tübingen, 72074 Tübingen, Germany

**Keywords:** CRP, platelet activation, degranulation, integrin, cytosolic Ca^2+^ concentration, caspase, phosphatidylserine translocation

## Abstract

Garcinol, an anti-inflammatory and anti-carcinogenic polyisoprenylated benzophenone isolated from Garcinia plants, stimulates tumor cell apoptosis and suicidal erythrocyte death, but supports the survival of hepatocytes and neurons. The present study explored whether the substance influences platelet function and/or apoptosis. To this end, we exposed murine blood platelets to garcinol (33 µM, 30 min) without and with activation by collagen-related peptide (CRP) (2–5 µg/mL) or thrombin (0.01 U/mL); flow cytometry was employed to estimate cytosolic Ca^2+^-activity ([Ca^2+^]_i_) from Fluo-3 fluorescence, platelet degranulation from P-selectin abundance, integrin activation from αIIbβ3 integrin abundance, caspase activity utilizing an Active Caspase-3 Staining kit, phosphatidylserine abundance from annexin-V-binding, relative platelet volume from forward scatter, and aggregation utilizing staining with CD9-APC and CD9-PE. As a result, in the absence of CRP and thrombin, the exposure of the platelets to garcinol did not significantly modify [Ca^2+^]_i_, P-selectin abundance, activated αIIbβ3 integrin, annexin-V-binding, cell volume, caspase activity, and aggregation. Exposure of platelets to CRP or thrombin was followed by a significant increase of [Ca^2+^]_i_, P-selectin abundance, αIIbβ3 integrin activity, annexin-V-binding, caspase activity, and aggregation, as well as significant cell shrinkage. All effects of CRP were strong and significant; those of thrombin were only partially and slightly blunted in the presence of garcinol. In conclusion, garcinol blunts CRP-induced platelet activity, apoptosis and aggregation.

## 1. Introduction

Garcinol, a polyisoprenylated benzophenone [1,2] isolated from *Garcinia cambogia* [2] or *Garcinia indica* [1,3,4], counteracts oxidative stress and inflammation [3]. Garcinol is effective against several malignancies [1,3] and favorably influences a variety of further clinical disorders such as cardiovascular disease, diabetes, gastric ailments, liver injury, allergy, and neurodegeneration [1,3,5,6]. Garcinol is effective against cancer in part by stimulating apoptosis [7,8,9,10], an effect paralleled by and presumably in part due to down-regulation of Akt [11], NFκB [12] and STAT3 [13], as well as activation of caspase 3 [14]. Garcinol has further been shown to counteract lipid peroxidation [2] and lipoxygenase activity [15]. Garcinol further stimulates the suicidal death of erythrocytes or eryptosis, an effect paralleled by oxidative stress and Ca^2+^ entry [16]. On the other hand, the putative effect on liver injury [6] and neurodegeneration [5] was attributed to inhibition of apoptosis.

Akt [17], NFκB [18,19], STAT3 [20], and caspases [21] are involved in generation, activation and apoptosis of blood platelets which contribute to primary hemostasis following vascular injury and by the same token contribute to the pathophysiology of acute thrombotic occlusion [22,23]. Disordered platelet function contributes to the pathophysiology of arterial thrombosis, vascular inflammation and atherogenesis [23,24]. Activation of platelets could be accomplished by an increase of cytosolic Ca^2+^ concentration ([Ca^2+^]_i_) [25] due to Ca^2+^ release from intracellular stores [26] and subsequent activation of Ca^2+^ release-activated channel Orai1 in the plasma membrane [25,27,28,29]. Caspase activation triggers platelet apoptosis paralleled by cell shrinkage and cell membrane scrambling with phosphatidylserine translocation to the cell surface [30,31].

To the best of our knowledge, an effect of garcinol on platelet activation and apoptosis has never been shown.

The present study thus explored whether garcinol influences platelet function and apoptosis prior to and following activation by CRP.

## 2. Results

The present study aimed to define the impact of garcinol on activation and apoptosis of blood platelets. To this end, murine platelets were isolated from wild type mice and incubated in Tyrode-HEPES buffer without or with activation by CRP in the absence and presence of garcinol.

**Figure 1:**
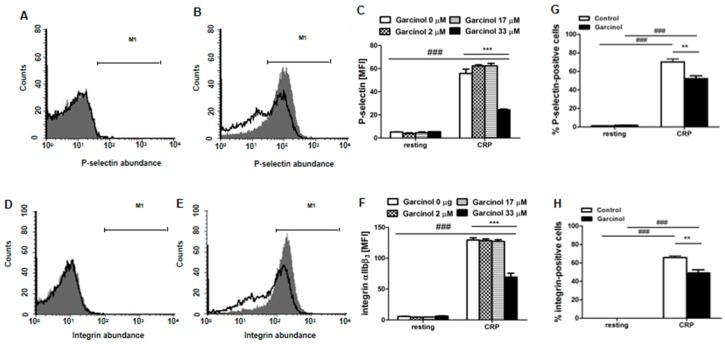
Garcinol-sensitive CRP-induced platelet degranulation and integrin α_IIb_β_3_ activation. (**A**,**B**) Original histogram overlays of P-selectin-related fluorescence in murine platelets without (**A**) and with (B) a 15 min CRP (2 µg/mL) treatment without (grey areas) and with (black lines) presence of garcinol (33 µM, 30 min). (**C**) Arithmetic means ± SEM (*n* = 4) of the P-selectin-related fluorescence (arbitrary units) in murine platelets without (left bars) and with (right bars) a 15-min CRP treatment (2 µg/mL) in the presence of 0–33 µM garcinol. (**D**,**E)** Original histogram overlays of activated α_IIb_β_3_ integrin-related fluorescence in murine platelets without (D) and with (E) a 15-min CRP (2 µg/mL) treatment without (grey areas) and with (black lines) presence of garcinol (33 µM, 30 min). (**F**) Arithmetic means ± SEM (*n* = 4) of activated α_IIb_β_3_ integrin-related fluorescence (arbitrary units) in murine platelets without (left bars) and with (right bars) a 15-min CRP treatment (2 µg/mL) in the presence of 0–33 µM garcinol. (**G**,**H**) Arithmetic means ± SEM (*n* = 4) of the percentage of P-selectin-positive (G) and of activated α_IIb_β_3_ integrin-positive (H) murine platelets in the absence (white bars) and presence (black bars) of 33 µM garcinol without (left bars) and with (right bars) a 15-min CRP treatment (2 µg/mL). ### (*p* < 0.001) indicates statistically significant difference from the absence of CRP; ** (*p* < 0.01) and *** (*p* < 0.001) indicate statistically significant difference from absence of garcinol.

Platelet degranulation was estimated from the increase of P-selectin abundance at the platelet surface, which was determined utilizing specific antibodies and flow cytometry. As illustrated in Figure 1A,C, without activation by CRP, the P-selectin abundance was negligible at the platelet surface and not significantly modified by garcinol (2–33 µM) treatment. CRP significantly increased P-selectin abundance, an effect significantly blunted in the presence of 33 µM garcinol (Figure 1B,C). Lower concentrations of garcinol (2 and 17 µM) did not significantly modify the effect of CRP on P-selectin abundance.

Similarly, the abundance of active integrin α_IIb_β_3_ at the platelet surface was in the absence of CRP negligible (Figure 1D,F) and not significantly modified by garcinol (2–33 µM) treatment. The abundance of active integrin α_IIb_β_3_ was significantly increased by CRP treatment. Again, the effect of CRP was significantly blunted in the presence of 33 µM garcinol (Figure 1E,F), but not in the presence of 2 and 17 µM garcinol.

**Figure 2:**
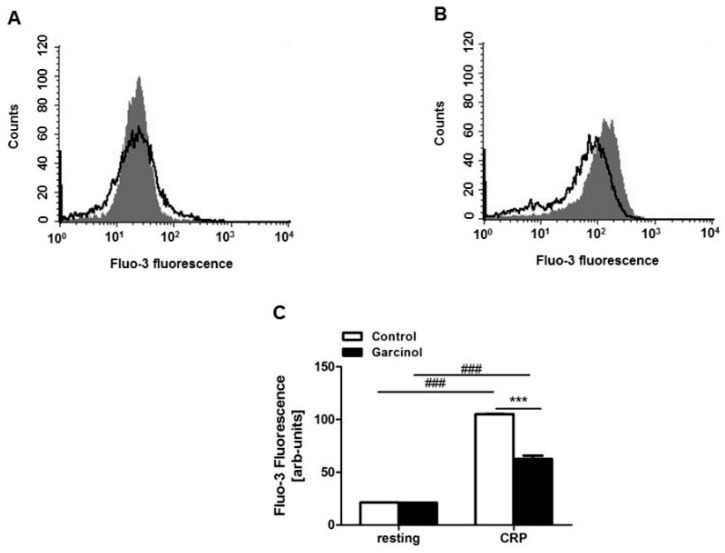
Garcinol-sensitive CRP-induced increase of cytosolic Ca^2+^ concentration. (**A**,**B**) Original histogram overlays of Fluo-3 fluorescence reflecting cytosolic Ca^2+^ activity in murine platelets without (A) and with (B) a 150 s treatment with CRP (2 µg/mL) without (grey areas) and with (black lines) presence of garcinol (33 µM, 30 min). (**C**) Arithmetic means ± SEM (*n* = 4) of Fluo-3 fluorescence reflecting cytosolic Ca^2+^ activity in murine platelets without (left bars) and with (right bars) a 150 s CRP treatment in the absence (white bars) and presence (black bars) of 33 µM garcinol. ### (*p* < 0.001) indicates statistically significant difference from absence of CRP; *** (*p* < 0.001) indicates statistically significant difference from absence of garcinol.

Cytosolic Ca^2+^ concentration ([Ca^2+^]_i_) was determined utilizing Fluo-3 fluorescence. As illustrated in Figure 2A,C, without activation by CRP [Ca^2+^]_i_ was similarly low in the absence and presence of garcinol. The histogram may, however, point to some minor increase of the fluorescence variability following garcinol treatment of resting blood platelets (Figure 2A). In any case, activation by CRP was followed by a significant increase of [Ca^2+^]_i_ in platelets, an effect significantly blunted in the presence of garcinol (Figure 2B,C).

**Figure 3:**
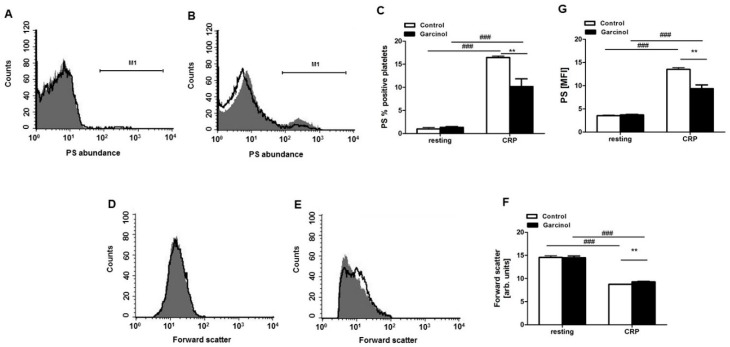
Garcinol-sensitive CRP-dependent cell membrane scrambling and cell shrinkage. (**A**,**B**) Original histogram overlays of annexin-V-binding reflecting phosphatidylserine abundance at the surface of murine platelets without (A) and with (B) a 10-min treatment with CRP (5 µg/mL) without (grey areas) and with (black lines) presence of garcinol (33 µM, 30 min). (**C**) Arithmetic means ± SEM (*n* = 4) of the percentage of annexin-V-binding murine platelets in the absence (white bars) and presence (black bars) of 33 µM garcinol without (left bars) and with (right bars) a 10 min CRP treatment (5 µg/mL). (**D**,**E**) Original histogram overlays of forward scatter reflecting cell volume of murine platelets without (D) and with (E) a 10-min treatment with CRP (5 µg/mL) without (grey areas) and with (black lines) presence of garcinol (33 µM, 30 min). (**F**) Arithmetic means ± SEM (*n* = 4) of forward scatter reflecting cell volume of murine platelets without (left bars) and with (right bars) a 10-min CRP treatment (5 µg/mL) in the absence (white bars) and presence (black bars) of 33 µM garcinol. (**G**) Arithmetic means ± SEM (*n* = 4) of annexin-V-binding in murine platelets without (left bars) and with (right bars) a 10-min CRP (5 µg/mL) treatment in the absence (white bars) and presence (black bars) of 33 µM garcinol. ### (*p* < 0.001) indicates statistically significant difference from absence of CRP; ** (*p* < 0.01) indicates statistically significant difference from absence of garcinol.

Phosphatidylserine abundance was estimated from annexin-V-binding. As illustrated in Figure 3A,C, the percentage of annexin-V positive platelets was again negligible in the absence of CRP, irrespective of the presence or absence of 33 µM garcinol. CRP significantly enhanced the percentage of annexin-V binding platelets, an effect again significantly blunted in the presence of 33 µM garcinol (Figure 3B,C).

Platelet volume was estimated from forward scatter, which was determined by flow cytometry. As illustrated in Figure 3D,F, in the absence of CRP, platelet volume was similar in the absence and presence of 33 µM garcinol. Activation of the platelets by CRP was followed by a marked decrease of forward scatter, an effect slightly but significantly blunted in the presence of 33 µM garcinol (Figure 3E,F).

**Figure 4:**
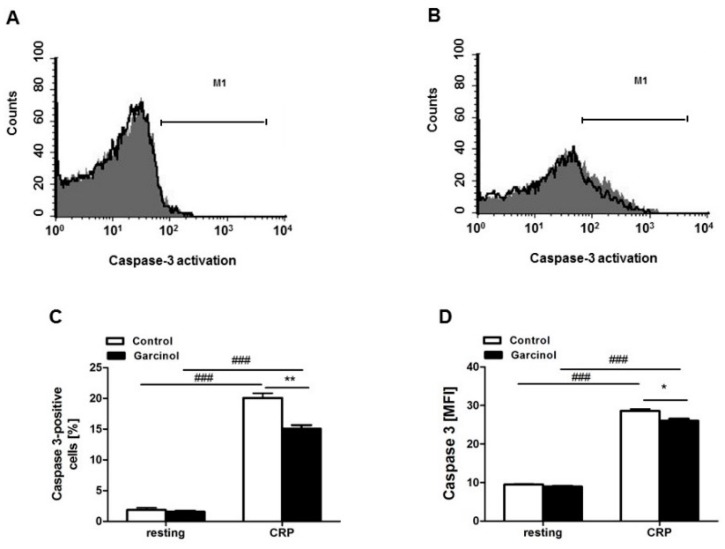
Garcinol-sensitive CRP-induced increase of caspase-3 activity. (**A**,**B**) Original histogram overlays of fluorescence reflecting caspase-3 activity in murine platelets without (A) and with (B) a 10-min treatment with CRP (5 µg/mL) without (grey areas) and with (black lines) presence of garcinol (33 µM, 30 min). (**C**) Arithmetic means ± SEM (*n* = 4) of the percentage of caspase-3-FITC-positive murine platelets in the absence (white bars) and presence (black bars) of 33 µM garcinol without (left bars) and with (right bars) a 10-min CRP treatment (5 µg/mL). (**D**) Arithmetic means ± SEM (*n* = 4) of caspase-3-FITC-related fluorescence of murine platelets in the absence (white bars) and presence (black bars) of 33 µM garcinol without (left bars) and with (right bars) a 10-min CRP treatment (5 µg/mL). ### (*p* < 0.001) indicates statistically significant difference from absence of CRP, * (*p* < 0.05); ** (*p* < 0.01) indicate statistically significant difference from absence of garcinol.

A kit has been used for the detection of activated caspase 3. As illustrated in Figure 4A,C, without activation by CRP, caspase activity was negligible, irrespective of the presence of garcinol. CRP significantly enhanced caspase activity, an effect again significantly blunted in the presence of 33 µM garcinol (Figure 4B–D). 

**Figure 5:**
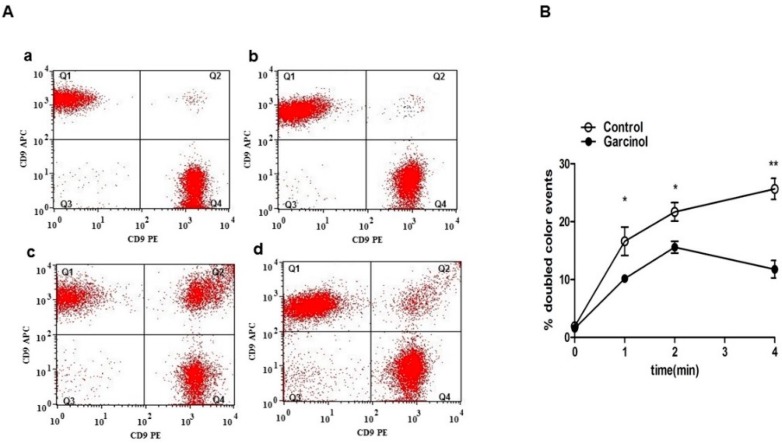
Garcinol-sensitive CRP-induced platelet aggregation. (**A**) Original dot blots reflecting platelet aggregation without (**a**,**c**) and with (**b**,**d**) prior garcinol (33 µM) treatment, and subsequent treatment with CRP (2 µg/mL) for 0 min (a,b) and 4 min (c,d). (**B**) Arithmetic means ± SEM (*n* = 4) of platelet aggregation without (white circles) and with (black circles) prior garcinol (33 µM) treatment as a function of time after addition of CRP (2 µg/mL). (*p* < 0.05) and ** (*p* < 0.01) indicates a statistically significant difference from absence of garcinol.

To elucidate the effect of CRP and garcinol on platelet aggregation, platelets were labeled with two distinct dyes and the coincidence of the two dyes (number of dots in Q2) estimated by flow cytometry. As illustrated in Figure 5Aa,Ab,B, without activation by CRP platelet aggregation was similarly low in the absence and presence of 33 µM garcinol. CRP treatment significantly increased platelet aggregation as reflected by the number of dots in Q2 (Figure 5Ac,B). The effect was significantly blunted by 33 µM garcinol (Figure 5Ad,B).

**Figure 6:**
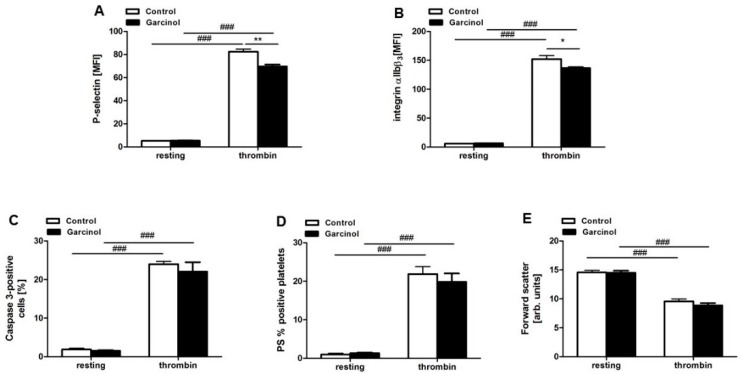
Effect of thrombin on P-selectin surface abundance, active integrin α_IIb_β_3_ abundance, caspase-3 activity, cell membrane scrambling, and cell volume in absence and presence of garcinol. Arithmetic means ± SEM (*n* = 4) of (**A**) P-selectin surface abundance, (**B**) active integrin α_IIb_β_3_ abundance, (**C**) caspase-3 activity, (**D**) annexin-V-binding, and (**E**) forward scatter prior to (resting) or 10–15 min following treatment with 0.01 U/mL thrombin in the absence (white bars) and presence (black bars) of garcinol (33 µM, 30 min). ### (*p* < 0.001) indicates statistically significant difference from absence of thrombin; * (*p* < 0.05) and ** (*p* < 0.01) indicate statistically significant difference from absence of garcinol.

An additional series of experiments explored whether garcinol similarly interferes with the effect of thrombin on blood platelets. As a result, thrombin (0.01 U/mL) treatment was followed by a sharp increase of P-selectin surface abundance (Figure 6A) of active integrin α_IIb_β_3_ abundance (Figure 6B), of caspase-3 activity (Figure 6C) and percentage of annexin-V binding platelets (Figure 6D), effects only slightly blunted by garcinol (33 µM). The effect of garcinol reached statistical significance only at the effect of thrombin on P-selectin surface abundance and active integrin α_IIb_β_3_ abundance. Thrombin (0.01 U/mL) treatment was further followed by platelet shrinkage (Figure 6E), an effect not significantly modified by treatment with garcinol (33 µM).

## 3. Discussion

The present observations disclose a novel powerful inhibitor of platelet activation and apoptosis. Treatment of murine blood platelets by garcinol significantly blunts the effect of CRP on [Ca^2+^]_i_, P-selectin abundance, α_IIb_β_3_ integrin activity, annexin-V-binding, cell volume, caspase activity, and aggregation. In the absence of CRP, garcinol had no appreciable effect on those parameters.

The observed effects of garcinol on platelet activation and apoptosis are at least in part due to a blunting of the CRP-induced increase of cytosolic Ca^2+^ activity ([Ca^2+^]_i_), a key event in platelet activation [18,28]. Excessive increase of [Ca^2+^]_i_ with subsequent over-activity of platelets fosters the development of arterial thrombosis [32]. An increase of [Ca^2+^]_i_ is further a powerful stimulator of cell membrane phospholipid scrambling with translocation of phosphatidylserine to the platelet surface [24,33,34,35]. Phosphatidylserine exposure at the platelet surface is part of the pro-coagulant function of platelets and thus participates in the orchestration of hemostasis [36]. Moreover, phosphatidylserine-exposing platelets are bound to and engulfed by macrophages [37].

The present paper did not address the signaling accounting for the effect of garcinol on [Ca^2+^]_i_. In tumor cells, garcinol is in part effective by down-regulation of Akt [11] and NFκB [12]. Both, Akt [17] and NFκB [18,19] participate in the regulation of platelet function. NFκB is known to up-regulate Orai1 [18], the major Ca^2+^ entry pathway into platelets [25,27,28,29]. However, it must be kept in mind that mature platelets lack nuclei and their function and survival could not be regulated by transcription.

Garcinol had negligible or no effects on the stimulation of platelets by thrombin. The signaling of thrombin differs from that of CRP. CRP is effective through (GP)VI-dependent signaling pathway [38], whereas thrombin is effective through G-protein coupled receptors [39].

In view of the present data, it is tempting to speculate that garcinol and related substances may be employed for the inhibition of platelet activation in the prophylaxis and/or treatment of thrombosis. Garcinol is a food additive which could be administrated to patients [40]. It must be kept in mind, though, that the present in-vitro studies in murine platelets cannot be translated without reservations into in-vivo effects on functions of human platelets. Clearly, additional experimental efforts are needed to define the effect of garcinol on platelet function in patients.

## 4. Conclusions

In isolated murine platelets, garcinol is a powerful inhibitor of CRP-induced increase of P-selectin abundance, α_IIb_β_3_ integrin activity, annexin-V-binding, caspase activity, and aggregation; and thus, it counteracts platelet activation and apoptosis. Garcinol is at least in part effective by increasing [Ca^2+^]_i_. In contrast, garcinol is only a weak inhibitor of thrombin-induced platelet stimulation. Additional effort is needed defining in vivo garcinol effects prior to considering the substance for prophylaxis/treatment of thrombosis in patients.

## 5. Materials and Methods 

### 5.1. Mice

All animal experiments were conducted according to the German law for the welfare of animals and were approved by the authorities of the state of Baden-Württemberg (Regierungspräsidium Tübingen according to §4, 19 December 2011). Experiments were performed with blood platelets isolated from wild type mice. The mice had free access to water and control chow (Ssniff, Soest, Germany) [40,41,42,43,44,45,46].

### 5.2. Preparation of Mouse Platelets

Platelets were prepared as described previously [40,41,42,43,44,45,46]. Platelets were obtained from 10- to 12-week-old mice of either sex. The mice were anesthetized, and 800 µL blood was drawn from the retro-orbital plexus into tubes with 200 µL acid-citrate-dextrose buffer before the mice were sacrificed [47]. Platelet-rich plasma (PRP) was obtained by centrifugation at 260 g for 5 min. Afterwards, PRP was centrifuged at 640 g for 5 min to pellet the platelets. Where necessary, apyrase (0.02 U/mL; Sigma-Aldrich) and prostaglandin I_2_ (0.5 µM; Calbiochem, Darmstadt, Germany) were added to the PRP to prevent the activation of platelets during isolation [48]. After two washing steps, the pellet of washed platelets was resuspended in modified Tyrode-HEPES buffer (pH 7.4, supplemented with 1 mM CaCl_2_). Where indicated, collagen-related peptide (Roche, Basel, Switzerland) or thrombin (Roche, Basel, Switzerland) were added at the indicated concentrations [49]. Platelets have been pre-incubated with 0–33 µM garcinol (R&D, Wiesbaden, Germany) for 30 min at 37 °C before stimulation.

### 5.3. Cytosolic Calcium

For the measurement of the cytosolic Ca^2+^ concentration, the platelet preparation was washed once in Tyrode buffer (pH 7.4), stained with 3 µM Fluo-3AM (Biotinium, USA) in the same buffer and incubated at 37 °C for 30 min. Following the indicated experimental treatment, relative fluorescence was measured utilizing a BD FACS Calibur (BD Biosciences, Heidelberg, Germany) [41,42,43,44,45,46,50].

### 5.4. P-Selectin and Activated Integrin Abundance

Fluorophore-labeled antibodies were utilized for the detection of P-selectin expression (Wug.E9-FITC) [51] and the active form of α_IIb_β_3_ integrin (JON/A-PE) [52]. Washed mouse platelets (1 × 10^6^) were suspended in modified Tyrode buffer (pH 7.4) containing 1 mM CaCl_2_ and antibodies (1:10 dilution) and subsequently subjected to the respective treatments and for the indicated time periods at room temperature (RT). The reaction was stopped by the addition of PBS and the samples were immediately analyzed on a BD FACSCalibur [24,41,42,43,44,45,46].

### 5.5. Phosphatidylserine Exposure and Forward Scatter

For the determination of phosphatidylserine exposure, the platelet preparation was centrifuged at 660 g for 5 min followed by washing once with Tyrode buffer (pH 7.4) with 1 mM CaCl_2_, staining with 1:20 dilution of Annexin-V-FITC (Mabtag, Friesoythe, Germany) in Tyrode buffer (pH 7.4) with 2 mM CaCl_2_ and incubation at 37 °C for 30 min. Annexin-V-binding reflecting surface exposure of phosphatidylserine was evaluated by flow cytometry utilizing a BD FACSCalibur. In parallel, the forward scatter (FSC) of the platelets was determined by flow cytometry as a measure of platelet size [41,42,43,44,45,46,53].

### 5.6. Caspase-3 Activity

Caspase 3 activity was determined utilizing a CaspGlow Fluorescein Active Caspase-3 Staining kit from BioVision (Milpitas, CA, USA) according to the manufacturer’s instruction. Fluorescence intensity was measured at an excitation wavelength of 488 nm and an emission wavelength of 530 in a BD FACSCalibur (BD Biosciences, USA) [41,42,43,44,45,46].

### 5.7. Platelet Aggregation

Aggregation was determined utilizing flow cytometry as previously described [54]. To this end, platelets were labeled either with CD9-APC or with CD9-PE monoclonal antibodies (1:100 dilution, Abcam) for 15 min at room temperature. Following incubation, differently labeled platelet samples were washed twice, mixed 1:1, and incubated in 33 µM garcinol (R&D, Germany) for 30 min at 37 °C while shaking at 600 rpm for 10 min. Pre-incubated platelets were activated with 2 µg/mL CRP at 37 °C while shaking at 1000 rpm. At the indicated time points, samples were fixed by the addition of 0.5% paraformaldehyde (Carl Roth, Karlsruhe, Germany) in phosphate-buffered saline. The fixed samples were measured utilizing a BD FACSCalibur (BD Biosciences, Heidelberg, Germany). For quantification, a quadrant was set in the dot plot of respective channels on non-stimulated platelets [41,42,43,46,49].

### 5.8. Statistical Analysis 

Data are provided as means ± SEM; n represents the number of independent experiments. All data were tested for significance using ANOVA with Tukey’s test as post-test or unpaired student’s *t*-test as appropriate. Results with *p* < 0.05 were considered statistically significant [41,42,43,44,45,46].
